# Book Reviews

**Published:** 1871-12

**Authors:** 


### BIBLIOGRAPHICAL.

*Practical Therapeutics.*—By Edward John Warring, M. D., F. L. S., Member of the Royal College of Physicians, Fellow of Royal College of Surgeons, etc. Second American, from the Third London Edition. Lindsay & Blakiston, Publishers, Philadelphia. A new edition of this valuable work will prove of great service to the student and young practitioner, especially as it contains such new matter as notices of Chloral, Bromide of Mercury, Iodide of Methyl, Bichloride of Methylene, Protoxide of Nitrogen, etc., etc., and extended notices of other preparations whose claims as therapeutic agents have only lately been fully recognized, such as the Bromide of Potassium, Alkaline Hypophosphites Hyposulphites, etc., etc., etc. The medical properties, therapeutic uses and doses of the various medicinal agents in common use are clearly and accurately stated rendering the work one of practical utility and an excellent text book. Over thirty pages are devoted to a valuable index of diseases, asterisks denoting the paragraphs most deserving of attention.

---

*The Teeth and How to Save Them.*—By L. P. Meredith, M.D., D.D.S.,. J. B. Lippincott & Co. Publishers, Philadelphia. This work, the author announces in his preface, is for the purpose of opening the doors of dental knowledge to the people, and of giving such suggestions and information relative to its uses and abuses as may awaken a better appreciation. The work is well written and contains much useful knowledge for the public, and some of which would be of benefit to certain members of our profession also, especially those who have allowed their calling to leave them far in the distance.

The volume is illustrated by a number of wood cuts, and is gotten up in a neat, attractive style creditable to the publishers. The author has certainly succeeded in his efforts, and has happily condensed much valuable information in a small space.

---

*Treatment and Prevention of Decay of the Teeth.*—A Practical and Popular Treatise. By Robert Arthur, M.D., D.D.S., with thirty-eight illustrations. J. P. Lippincott & Co., Publishers, Philadelphia. The author of this work, in his preface remarks that he offers it as the result of a long and very careful study of the subject of which it treats.

As the opinions held by Dr. Arthur on the subject of "prevention of decay of the teeth," have often been misrepresented, the publication of this work will be gratifying to the profession of which he is so honored a member; and while all who read it may not agree with some of the suggestions advanced, yet the able manner in which they are discussed, will have the effect of causing the most skeptical to consider them more carefully than heretofore, now that their true nature is understood.

By the system of treatment described in this work, the author claims 1st, That the teeth may be more certainly preserved. 2nd, That the treatment required is much more simple than that at present relied upon; and 3d, That the pain usually attendant upon dental operations may be entirely avoided.

Chapter 1. Is devoted to a brief discussion of the Anatomy of the Teeth-Enamel, Dentine, "Nerve," etc. Chapter II. treats of the causes and nature of decay—that the enamel is not always a protection even when perfectly formed, especially on surfaces in contact, offering as an example, cases where the crowns are completely worn down to a level with the gums by the friction of mastication, and the dentine remaining free of decay—that the enamel, from its hard, flinty character, is evidently intended to bear the friction to which it is subjected, but that it has no capacity of resisting attacks of decay under favoring circumstances. Chapter, III. is devoted to the "Treatment of Decay," and refers to the very great advances which have been made in the methods of using gold—the good effects of separating teeth in close contact to prevent decay, from the fact that teeth standing apart, are as a general rule exempt from its attacks, and thus obviate the necessity for filling them afterwards.

Chapter IV. is devoted to the importance of preserving the first permanent molars, and recommends that "as soon as the first permanent molars are formed, the temporary molars in contact with them should be cut away so that the permanent molars may be isolated." Chapter V. is devoted to the "treatment of the incisor teeth," where decay rarely occurs except upon the approximal surfaces, when these surfaces are in contact, and contends that as the necessary attention is rarely given on the part of the patient, superficial decay can be more effectually arrested by a permanent separatien, that by the operation of filling.

Chapters VI. and VII. treat of the "bicuspid and molar teeth which are equally as liable to decay as incisors," and describes in detail the means of prevention of decay, advocating the method of separating bicuspids and molars, even when perfectly sound, as a preventive to caries, and contending that the relative liability of the different classes of teeth to decay is about the same. Many will differ from the author as to this method of treatment being the proper one, and take exceptions to the earnest effort he makes to prove the correctness of the opinion he holds upon this subject.

The following table is given, after a very careful estimate of numerous cases examined, as a close approximation to the relative liability of the teeth to decay upon the proximate surfaces when all the usual efforts are made to treat it as it occurs:

Table of 1000 cases of Decay of Proximate Surfaces:

| | | per cent. |
|---|---|---|
| Central incisors, | 121 | 12.1 |
| Lateral " | 90 | 9.0 |
| Canines | 80 | 8.0 |
| 1st Bicuspids, | 172 | 1.72 |
| 2nd " | 156 | 1.56 |
| 1st Molars, | 245 | 2.45 |
| 2nd " | 113 | 1.13 |
| Wisdom Teeth, | 23 | 2.3 |

Chapter VIII. is devoted to the "prevention of decay of the teeth," the author contending that as a general thing, the bicuspid and molor teeth will decay if the proximate surfaces of the incisors be attacked by caries at an early age. He calls the system he proposes, that of a permanent separation, a preventive one, and says: "what I propose is *the separation of teeth closely in contact, which are of such frail character, and are exposed to such destructive influences, that their decay is inevitable, or has already occurred.*" Chapter IX. is devoted to "some of the more serious consequences of decay of the teeth," such as exposure of the nerve, alveolar abscess, and neuralgia. Chapter X. is devoted to the "practice of dentistry," in a thorough and faithful manner, and dwells upon the serious injuries resulting from imperfect treatment. This work will prove an important addition to the dental literature of our day, and is worthy of a careful perusal by every member of the profession.

